# Favorable effect of ripasudil use on surgical outcomes of microhook ab interno trabeculotomy

**DOI:** 10.1007/s00417-023-06040-1

**Published:** 2023-03-31

**Authors:** Mina Okuda, Sotaro Mori, Kaori Ueda, Mari Sakamoto, Sentaro Kusuhara, Yuko Yamada-Nakanishi, Makoto Nakamura

**Affiliations:** 1 Division of Ophthalmology, Department of Surgery, Kobe University Graduate School of Medicine, 7-5-1 Kusunoki-Cho, Chuo-Ku, Kobe, 650-0017 Japan; 2 Institute of Ophthalmology, University College London, London, UK

**Keywords:** Ripasudil hydrochloride hydrate, Schlemm’s canal surgery, *Ab interno* trabeculotomy, Rho kinase inhibitor, Propensity score analysis

## Abstract

**Purpose:**

We have previously demonstrated that prolonged use of glaucoma medications was associated with a poor surgical outcome of *ab interno* trabeculotomy (µTLO). Given that almost all types of glaucoma eye drop either enhance the drainage through the uveoscleral pathway or reduce aqueous humor production, we hypothesized that prolonged use of these medications might cause disuse atrophy of the conventional pathway. In contrast, ripasudil increases the conventional outflow and eventually shows a favorable outcome of µTLO. This study aimed to evaluate the effect of ripasudil use on µTLO outcomes.

**Method:**

The medical charts of 218 patients who underwent µTLO were analyzed retrospectively. We compared the 1-year outcome between ripasudil users versus nonusers by using propensity score matching. We set the covariates as age, sex, glaucoma types, preoperative intraocular pressure (IOP), the mean deviation values of visual field tests, the presence or absence of concomitant cataract surgery, trabecular meshwork incision range, the presence or absence of any glaucoma medication except ripasudil and duration of glaucoma medical therapy. Success was defined as a postoperative IOP between 5 and 21 mmHg, a ≥ 20% IOP reduction from baseline, and no additional glaucoma surgery at postoperative 1 year.

**Result:**

Fifty-seven patients each were allocated to the ripasudil users or nonusers. The 1-year success rates were 74% in ripasudil users and 51% in nonusers (*p* = 0.01). Kaplan‒Meier survival curves also showed that the ripasudil users had a higher survival distribution (*p* = 0.01).

**Conclusion:**

The patients who took ripasudil showed a favorable 1-year outcome of µTLO.

**Supplementary Information:**

The online version contains supplementary material available at 10.1007/s00417-023-06040-1.


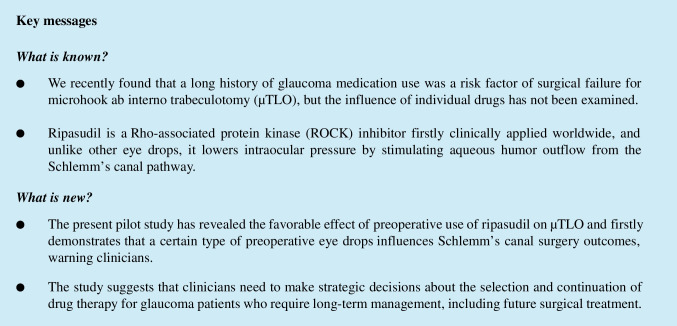


## Introduction

Glaucoma treatment has dramatically developed both medically and surgically. Ripasudil hydrochloride hydrate (ripasudil), which is a Rho kinase inhibitor, has emerged as a drug with a new mechanism and has been used for clinical practice in Japan and South Korea [1]. Other main types of glaucoma medications available in Japan promote drainage into the uveoscleral pathway or decrease aqueous humor production. In contrast, ripasudil increases the conventional trabecular meshwork—Schlemm’s canal outflow—based on its Rho kinase inhibitory action [2]. Another study showed that ripasudil induced morphological changes in type VI collagen of the juxtacanalicular tissue [3].

From a surgical viewpoint, minimally/micro invasive glaucoma surgery (MIGS) has become applicable for treating mild to moderate glaucoma patients. MIGS is classified into two categories: Schlemm’s canal surgeries and filtration surgeries such as PreserFlo and Xen. The former type of MIGS incises the trabecular meshwork, the main aqueous humor resistance of the trabecular outflow pathway via an ab interno and ab externo approach. Ab interno Schlemm’s canal surgery [4], sometimes called trabeculotomy [5], goniotomy [6] or goniectomy [7], includes iStent, Trabectome, Kahook Dual Blade, TrabEx + , gonioscopy-assisted transluminal trabeculotomy, Hydrus Microstent, and viscocanalostomy [8]. In Japan, *ab interno *trabeculotomy using a “microhook” device developed by Tanito (µTLO) [9] has gained enormous popularity for two reasons. First, µTLO shows cost-effectiveness because the microhook is reusable after sterilization. Second, our recent multicenter study has shown that µTLO has a similar surgical outcome to Trabectome, the pioneer of MIGS [10].

On the other hand, our recent study revealed that the prolonged use of glaucoma medications had a negative impact on postoperative outcomes of µTLO [11]. We speculate that this may be attributable to disuse atrophy of the trabecular outflow (conventional) pathway in patients who took long-term glaucoma medications, given their mechanisms of action as mentioned above.

Taking the pharmacological action of ripasudil into consideration, it is reasonable to hypothesize that ripasudil, unlike other medications, may not cause disuse atrophy of the trabecular meshwork (Schlemm’s canal pathway), which could rather show a favorable surgical outcome of µTLO. The purpose of this study was to investigate the effect of preoperative ripasudil administration on µTLO surgical outcomes at 1 year.

## Method

### Subjects

The medical charts of all adult patients (≥ 20 years old) who underwent µTLO between February 2017 and April 2021 in Kobe University Hospital were reviewed retrospectively. Patients who had a history of glaucoma surgery, including laser surgery, were excluded. When both eyes were operated on, the first eye was selected. We thus analyzed 218 eyes of 218 patients in total.

### Clinical characteristics

The information collected was as follows: age, sex, glaucoma types, intraocular pressure (IOP), glaucoma drug score, best-corrected decimal visual acuity (VA), the mean deviation values of Humphrey visual field tests (MD), corneal endothelial cell density (ECD), the presence or absence of any glaucoma medication at the time of surgery, the overall duration of glaucoma medical therapy, and whether antithrombotic drugs were used preoperatively.

The glaucoma drug score was counted as one point for each eye drop but two points for combined eye drops and oral administration of carbonic anhydrase inhibitors [10–14]. We also collected perioperative information about the presence or absence of concomitant cataract surgery and trabecular meshwork incision range (one or two quadrants). The detailed surgical technique of µTLO was described previously [11, 12, 14]. We analyzed IOP and glaucoma drug scores preoperatively and 1 week, 1, 3, 6, 9, and 12 months postoperatively and VA, MD, and ECD preoperatively. For eyes that required additional surgery within 1 year after the initial surgery, we imputed postoperative parameters using the last observation carried forward method. We counted the number of patients who had hyphema and transiently elevated IOP as early surgery-related complications. The presence of layered hyphema was regarded as hyphema in this study. The term “transiently elevated IOP” refers to postoperative IOP exceeding the preoperative value despite the use of the same glaucoma drugs as preoperatively [10, 11, 14]. After surgery, the choice of postoperative medications is based on each patient’s target IOP. Surgeons will resume appropriate drugs referring to their preoperative medications.

### Data analysis

Surgical success was defined as meeting these three criteria, as follows: (1) IOP within 5–21 mmHg, (2) reduction in IOP by at least 20% from the preoperative IOP, and (3) no additional glaucoma surgery [10–12, 14]. Surgical failure was marked when additional glaucoma surgeries were conducted or the IOP was out of the above mentioned range on two consecutive time points after 1 month postoperatively, and the earlier point was defined as the date of failure. The distribution of surgical success between the ripasudil users and nonusers was compared using a log-rank test based on the Kaplan‒Meier survival curve. Ripasudil users were defined as individuals who took the drug at the time of surgery. We also compared the preoperative and postoperative parameters between the two groups by the Mann‒Whitney *U* test for continuous variables and the chi-square test or Fisher’s exact test for binary variables. A *p*-value < 0.05 was considered statistically significant. Statistical analysis was performed using MedCalc (version 20.015, MedCalc Software, Mariakerte, Belgium).

### Propensity score matching analysis

When we compared the perioperative characteristics between the ripasudil users and nonusers, the raw data showed significant differences in the proportions of patients with steroid-induced glaucoma, the preoperative IOP, and the glaucoma drug score (Supplemental Table 1). Previous reports showed that steroid-induced glaucoma had a higher surgical success rate than primary open-angle glaucoma [15, 16]. Preoperative high IOP was a risk factor for surgical failure in Schlemm’s canal surgery [17–20]. Comparing patients from different backgrounds likely induces biases. To cope with this problem, we used a propensity score analysis. A propensity score was defined as the conditional probability of two groups given the observed covariates. The covariates used in building the propensity score were age, sex, glaucoma types, preoperative IOP, MD, the presence or absence of combined cataract surgery, incision range, the presence or absence of any glaucoma medications except ripasudil at the time of surgery, and overall duration of glaucoma medical therapy. We performed a one-to-one matching analysis between the two groups based on the estimated propensity score of each patient using a logistic regression model. A caliper width of 0.2 of the standard deviation of the propensity score was used for the one-to-one matching analysis [10].

## Results

Table 1 shows the preoperative characteristics after propensity score matching between the ripasudil users and nonusers. After one-to-one matching, 57 patients each were allocated into the two groups. Propensity score matching eliminated the significant differences in the glaucoma types and preoperative IOP that existed prior to the matching. There was one eyedrop difference in the median glaucoma drop score between the ripasudil users and nonusers (median 5.0 vs. 4.0; *p* < 0.001).

**Table 1:** Patient demographics after propensity score matching

		Ripasudil users *N* = 57		Ripasudil nonusers *N* = 57		*P* value
Age, years	Median (IQR)	71.0	(66, 77)	69.0	(60, 75)	0.46†
Male	*n* (%)	33	(56)	29	(51)	0.45*
Glaucoma type						
POAG	*n* (%)	33	(58)	25	(44)	0.14*
XFG	*n* (%)	8	(14)	16	(28)	0.07*
Steroid G	*n* (%)	4	(7)	1	(2)	0.36‡
Others	*n* (%)	12	(21)	15	(26)	0.51*
Preoperative IOP, mmHg	Median (IQR)	26.0	(21.0, 34.0)	26.0	(21.0, 31.0)	0.57†
Preoperative glaucoma drug score	Median (IQR)	5.0	(5.0 6.0)	4.0	(3.0, 6.0)	< 0.001†
Preoperative HVF MD value, dB	Median (IQR)	− 12.94	(− 20.15, − 5.89)	− 11.74	(− 19.73, − 8.04)	0.69†
Preoperative logMAR visual acuity	Median (IQR)	0.05	(− 0.08, 0.15)	0.00	(− 0.08, 0.30)	0.39†
Preoperative ECD, cells/mm^2^	Median (IQR)	2532	(2201, 2829)	2532	(2315, 2770)	0.76†
Combined cataract surgery	*n* (%)	27	(47)	23	(40)	0.45*
Incision range (1quadrant)	*n* (%)	51	(90)	46	(81)	0.19*
Overall duration of glaucoma medical therapy, years	Median (IQR)	4.6	(0.8 12.0)	5.8	(1.4, 11.0)	0.67†
Antithrombotic drug use	*n* (%)	11	(19)	8	(14)	0.45*

*IQR*, interquartile range; *POAG*, primary open angle glaucoma; *XFG*, exfoliation glaucoma; *steroid G*, steroid-induced glaucoma; *IOP*, intraocular pressure; *HFV*, Humphrey visual field; *MD*, mean deviation; *ECD*, corneal endothelial density; *1quadrant*, the patient who had 120° incision of trabecular meshwork;*, chi-squared test; †, Mann–Whitney *U* test; ‡, Fisher’s exact test

The glaucoma medications used preoperatively in the two groups after matching adjustments are listed in Table 2. We classified the types of glaucoma medications according to the Japan Glaucoma Society Guidelines for Glaucoma [21]. Prostanoid receptor analogs were administered in 97% of the patients of both groups. The other three types of eyedrops (β-blockers, carbonic dehydrate inhibitors, and an α2 adrenergic agonist) followed the proportion of use in these patients. Few patients used the remaining three types (an α1 adrenergic antagonist, an ion-channel opener, and a parasympathomimetic). There was no significant difference in the proportion of each glaucoma eye drop except for ripasudil (*p* < 0.0001).

**Table 2:** The number of patients who were preoperatively administered each glaucoma drug

		Ripasudil users *N* = 57		Ripasudil nonusers *N* = 57		*P* value
Eye drops						
Prostanoid receptor analogs	*n* (%)	55	(97)	55	(97)	1.00*
β-blockers	*n* (%)	48	(84)	51	(90)	0.41*
Carbonic anhydrase inhibitors	*n* (%)	47	(83)	53	(93)	0.09*
α2 adrenergic agonist	*n* (%)	45	(79)	40	(70)	0.28*
α1 adrenergic antagonist	*n* (%)	0	(0)	4	(7)	0.12†
Ion-channel opener	*n* (%)	0	(0)	0	(0)	1.00†
Parasympathomimitic	*n* (%)	0	(0)	0	(0)	1.00†
Rho kinase inhibitor	*n* (%)	57	(100)	0	(0)	< 0.0001†
Oral medicine						
Acetazolamide	*n* (%)	22	(39)	22	(39)	1.00*

^*^, Chi-squared test; †, Fisher’s exact test

**Fig. 1:**
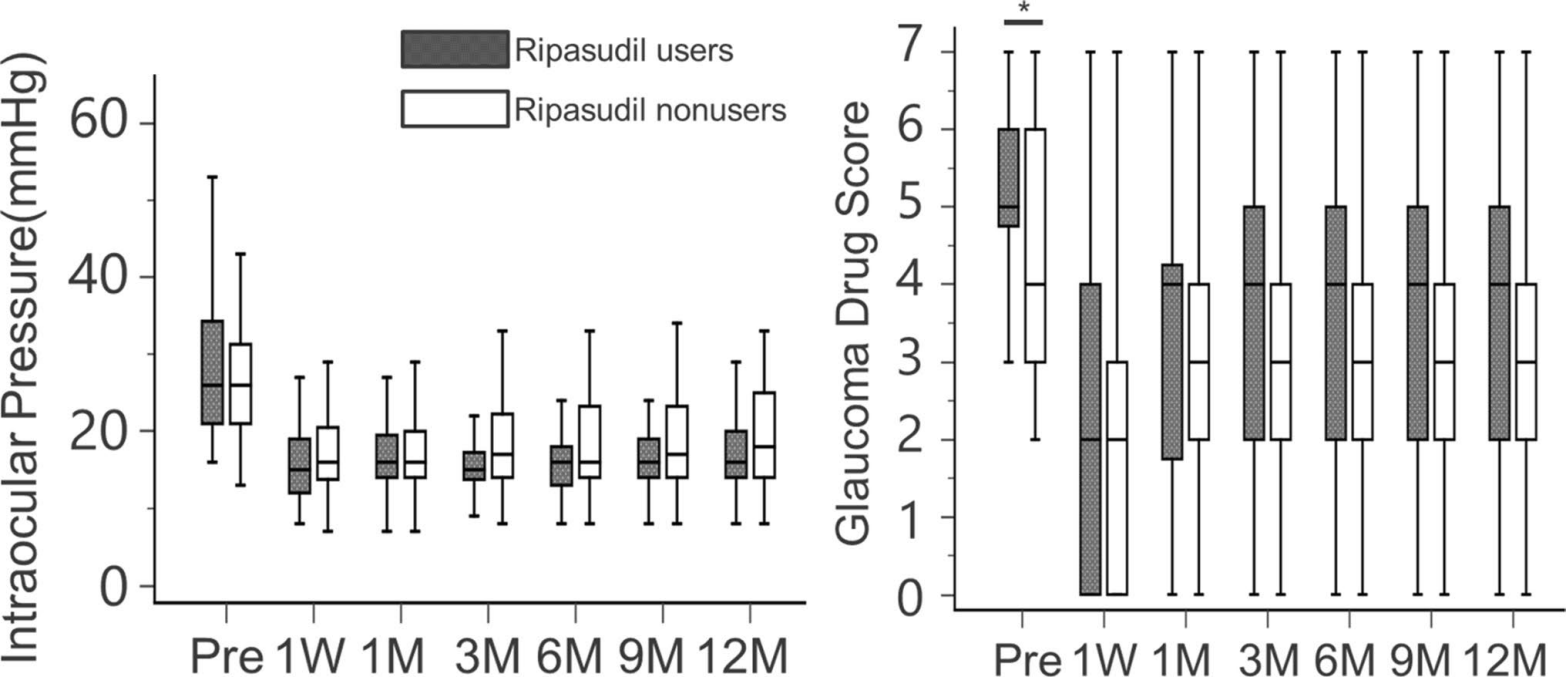
Box-and-whisker charts for time course changes of intraocular pressure and glaucoma drug score. Pre, preoperative; 1W, 1 week; 1 M, 1 month; 3 M, 3 months; 6 M, 6 months; 9 M, 9 months; 12 M, 12 months postoperatively; *, *p* < 0.05; †, Mann–Whitney *U* test

The time-course changes in the IOP and glaucoma drug scores in the two groups are shown in Fig. 1. Both groups demonstrated significantly decreased postoperative IOP and glaucoma drug scores compared to the preoperative values at each time point (*p* < 0.001). The median postoperative IOP in the ripasudil users was lower than that in the nonusers, but a statistically significant difference was not found at any postoperative time points. The glaucoma drug score was also not different between the two groups at any point.

Table 3 summarizes the surgical outcomes of early surgery-related complications and 1-year results. There was no difference in early surgery-related complications between the two groups. There was no significant difference in IOP at 1 year postoperatively between the ripasudil users and nonusers (median 16.0 vs. 18.0 mmHg, *p* = 0.09). The ripasudil users accounted for a significantly lower proportion of additional glaucoma surgeries (9% vs. 28%, *p* = 0.01). The success rate of the ripasudil users was 74%, while that of the nonusers was 51% at postoperative 1 year. There was a significant difference in the 1-year success rate between the two groups (*p* = 0.01).

**Table 3:** Early surgery-related complications and one-year postoperative outcomes

		Ripasudil users *N* = 57		Ripasudil nonusers *N* = 57		*P* value
Layered hyphema	*n* (%)	17	(30)	17	(30)	1.00*
Transient increase in IOP	*n* (%)	15	(26)	21	(37)	0.23*
IOP, mmHg	Median (IQR)	16.0	(14.0, 20.0)	18.0	(14.0, 25.0)	0.09†
Glaucoma drug score	Median (IQR)	4.0	(2.0, 5.0)	3.0	(2.0, 4.0)	0.38†
Additional glaucoma surgeries	*n* (%)	5	(9)	16	(28)	0.01‡
Success rate	*n* (%)	42	(74)	29	(51)	0.01*

*IOP*, intraocular pressure; *IQR*, interquartile range; *, chi-squared test; †, Mann–Whitney *U* test; ‡, Fisher’s exact test

**Fig. 2:**
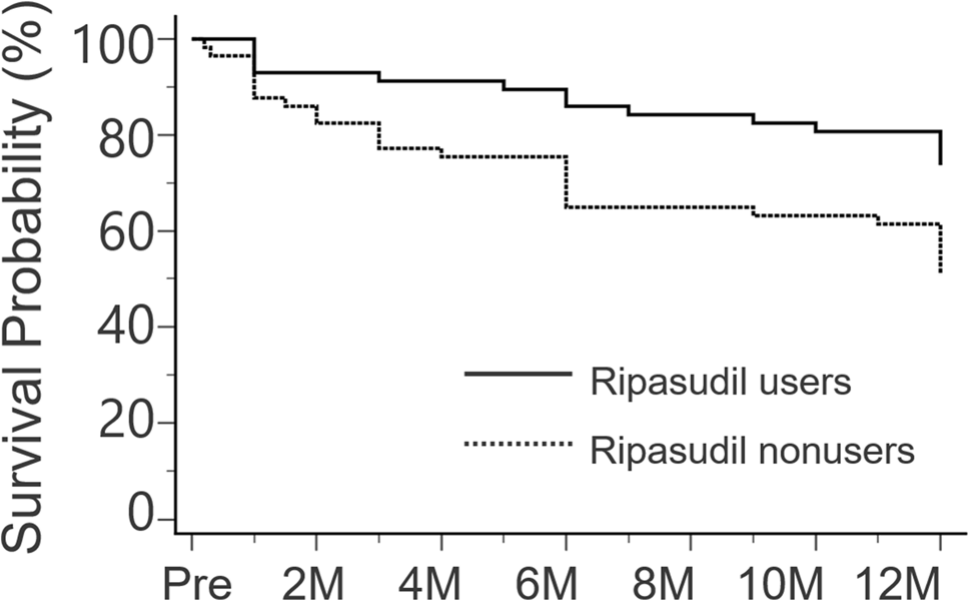
Kaplan–Meier survival curves for surgical success. There was a significant difference in the surgical success probability between the two groups (*p* = 0.01, log-rank test). Pre, preoperative; 2 M, 2 months; 4 M, 4 months; 6 M, 6 months; 8 M, 8 months; 10 M, 10 months; 12 M, 12 months postoperatively

Figure 2 shows the Kaplan‒Meier survival curves in the two groups. The log-rank test revealed that the ripasudil users had a significantly higher survival distribution (*p* = 0.01).

## Discussion

The present study elucidated that ripasudil users had significantly better IOP control 1 year after µTLO than nonusers. This is the first study to show that a certain type of preoperative eye drop influences the Schlemm’s canal surgery outcomes.

The glaucoma eye drops mainly used in Japan are classified into eight categories: prostanoid receptor analogs, β-blockers, carbonic anhydrase inhibitors, an α2 adrenergic agonist (brimonidine), an α1 adrenergic antagonist (bunazosin), an ion-channel opener (unoprostone isopropyl), a parasympathomimetic (pilocarpine), and a Rho kinase inhibitor (ripasudil). The prostanoid receptor analogs are subdivided into an EP2 receptor selective agonist (omidenepag isopropyl) and FP receptor agonists such as latanoprost, tafluprost, travoprost, and bimatoprost. As recommended by the 5th version of the Japan Glaucoma Society Guidelines for Glaucoma, prostanoid receptor analogs are deemed to be the first choice for glaucoma eye drops in Japan [21]. The second line is up to the preferences of physicians. Consequently, prostanoid receptor analogs were used in 97% of the patients in both the ripasudil users and nonusers in this study, while the proportion of other types of medication use was lower. Among these agents, ripasudil [2], an ion-channel opener [22], a parasympathomimetic [23], and an EP2 receptor selective agonist [24] are reported to increase the conventional outflow. In our study, no patients were administered an EP2 receptor selective agonist because this drug was approved in Japan quite recently (November 2018). The remaining three drugs are not prescribed as often in Japanese clinical practice due to their weak IOP-lowering effect. Therefore, the main glaucoma eye drops, except EP2 receptor selective agonist and ripasudil, may result in atrophy of the trabecular outflow pathway. It has been reported that Schlemm’s canal shrinks after trabeculectomy, a surgery for draining aqueous humor without using the physiological pathway [25].

We developed another hypothesis when we found an association between a long history of glaucoma medications and poor µTLO outcomes [11]. Eye drops contain various preservatives and additives, such as benzalkonium chloride (BAK), which might reduce the surgical success rate in µTLO. In fact, BAK is known to activate inflammatory mediators and induce apoptosis of trabecular meshwork cells in vivo [26]. Additionally, a similar mechanism has been reported in which BAK affects not only Schlemm’s canals but also subsequent collector channels [27]. Thus, we speculated that long-term exposure to BAK could contribute to reducing the surgical success rate in µTLO. However, ripasudil also contains BAK. Given the significant differences in the surgical outcomes between ripasudil users and nonusers, this scenario is unlikely.

While our study focused on the effect of µTLO in patients who did or did not receive preoperative ripasudil, it is possible that postoperative medications could have affected our results. Rho kinase inhibitors, including ripasudil, have been shown to inhibit fibroblast proliferation and reduce scarring after glaucoma surgery [28]. To investigate the potential impact of postoperative medications, we analyzed the postoperative use of ripasudil in our study population after matching (Supplemental Table 2) and compared the proportion of postoperative medications between the two groups (Supplemental Table 3). The presence or absence of postoperative medications did not significantly influence our results. Overall, the present study supports the use of preoperative ripasudil to enhance the efficacy of µTLO.

This study has several limitations. First, this is a retrospective single-center pilot study. Second, our speculation lacks histological evidence. Third, the onset and duration of ripasudil administration for each individual patient remain indeterminate. An examination of these specifics was unattainable due to prior physician-prescribed use of ripasudil. Such information may facilitate a more informed determination of the optimal time to administer ripasudil. Fourth, it may also be possible that the two groups have unintentional background biases. An assignment by propensity score has the disadvantage of equalizing only the known confounders but not any unknown background variables, which can hold bias. Almost all the current subjects were referred to our hospital, with the treatment being initiated by clinics. Whether ripasudil was prescribed essentially depended on the discretion of the clinic’s physicians; thus, there were no clear selection criteria for ripasudil administration. For example, the raw data (Supplemental Table 1) showed that the ripasudil users had a high proportion of steroid-induced glaucoma. This may be influenced by our previous report that ripasudil had a stronger IOP-decreasing effect in steroid-induced glaucoma than in primary open-angle or exfoliation glaucoma [29].

In conclusion, our study revealed that preoperative ripasudil use showed significantly better outcomes of µTLO at 1 year postoperatively. Given that ripasudil, unlike other major drugs, reduces IOP through the trabecular outflow pathway, this study may suggest that ripasudil does not induce disuse atrophy of this pathway.

## Supplementary Information

Below is the link to the electronic supplementary material.

Supplementary file1 (PDF 120 KB)

Supplementary file2 (PDF 68 KB)

Supplementary file3 (PDF 191 KB)

## Data Availability

The data that support the findings of this study are available from the corresponding author upon reasonable request.

## Materials availability

The data that support the findings of this study are available from the corresponding author upon reasonable request.
