# Supplementary material for: Favorable effect of ripasudil use on surgical outcomes of microhook ab interno trabeculotomy
Source: Graefes Arch Clin Exp Ophthalmol. 2023 Mar 31;261(9):2603–10. doi: 10.1007/s00417-023-06040-1 (PMC10432324; doi:10.1007/s00417-023-06040-1)
Supplement: Supplementary file 1 — Supplementary file1 (PDF 120 KB) [file 417_2023_6040_MOESM1_ESM.pdf]

Supplemental Table 1. Patient demographics of the study population before propensity score matching

			Ripasudil users n=125		Ripasudil nonusers n=93		p value
Age, yrs		median (IQR)	70.0	(60, 76)	69.0	(60, 75)	0.72†
Male		n (%)	70	(56)	47	(51)	0.42*
Glaucoma type	POAG	n (%)	53	(42)	51	(55)	0.07*
	XFG	n (%)	20	(16)	21	(23)	0.22*
	Steroid G	n (%)	14	(11)	1	(1)	<0.01‡
	Others	n (%)	38	(30)	20	(22)	0.14*
Preoperative IOP, mmHg		median (IQR)	28.0	(22.0, 35.0)	25.0	(21.0, 29.0)	<0.01†
Preoperative glaucoma drug score		median (IQR)	5.0	(5.0 6.0)	4.0	(3.0, 5.0)	<0.0001†
Preoperative HVF MD value, dB		median (IQR)	-11.37	(-18.75, -4.90)	-11.63	(-16.68, -7.15)	0.63†
Preoperative logMAR visual acuity		median (IQR)	0.00	(-0.08, 0.22)	0.00	(-0.08, 0.22)	0.93†
Preoperative ECD, cells/mm^2		median (IQR)	2577	(2222, 2878)	2591	(2354, 2795)	0.89†
Combined cataract surgery		n (%)	49	(39)	47	(51)	0.10*
Incision range (1quadrant)		n (%)	105	(84)	81	(87)	0.52*
Overall duration of glaucoma medical therapy, yrs		median (IQR)	4.6	(1.0, 10.8)	5.4	(1.5, 11.0)	0.46†
Antithrombotic drug use		n (%)	19	(15)	14	(15)	0.98*

IQR, Interquartile range; POAG, Primary open angle glaucoma; XFG, Exfoliation glaucoma; Steroid G, Steroid-induced glaucoma; IOP, intraocular pressure; HFV: Humphrey visual field; MD, Mean deviation; ECD, corneal endothelial density; 1-quadrant, the patient who had 120° incision of trabecular meshwork; *, Chi-squared test; †, Mann–Whitney U test; ‡, Fisher's exact test
