# Supplementary material for: Favorable effect of ripasudil use on surgical outcomes of microhook ab interno trabeculotomy
Source: Graefes Arch Clin Exp Ophthalmol. 2023 Mar 31;261(9):2603–10. doi: 10.1007/s00417-023-06040-1 (PMC10432324; doi:10.1007/s00417-023-06040-1)
Supplement: Supplementary file 2 — Supplementary file2 (PDF 68 KB) [file 417_2023_6040_MOESM2_ESM.pdf]

Supplemental Table 2. Comparison of surgical outcomes with and without ripasudil use one year after surgery.

	Postoperative Users (n=38)	Postoperative Nonusers (n=76)	p value
Surgical Success, n (%)	22 (58)	49 (64)	0.50*

(*, Chi-squared test)
