# Supplementary material for: Favorable effect of ripasudil use on surgical outcomes of microhook ab interno trabeculotomy
Source: Graefes Arch Clin Exp Ophthalmol. 2023 Mar 31;261(9):2603–10. doi: 10.1007/s00417-023-06040-1 (PMC10432324; doi:10.1007/s00417-023-06040-1)
Supplement: Supplementary file 3 — Supplementary file3 (PDF 191 KB) [file 417_2023_6040_MOESM3_ESM.pdf]

Supplemental Table 3. Comparison of postoperative glaucoma medications except for ripasudil in preoperative ripasudil users and nonusers.

		Ripasudil users n=57		Ripasudil nonusers n=57		p value
Prostanoid receptor analogs	n (%)	47	(82)	52	(91)	0.17*
β-blockers	n (%)	41	(72)	44	(77)	0.52*
Carbonic anhydrase inhibitors	n (%)	39	(68)	43	(75)	0.41*
α2 adrenergic agonist	n (%)	28	(49)	26	(46)	0.71*
α1 adrenergic antagonist	n (%)	0	(0)	2	(4)	0.50†
Ion-channel opener	n (%)	0	(0)	0	(0)	1.00†
Parasympathomimitic ※	n (%)	57	(100)	57	(100)	1.00*
Acetazolamide	n (%)	4	(7)	12	(21)	0.06†

※ Our institution uses parasympathomimetic for about 1 month postoperatively in all cases to prevent peripheral anterior synechia formation.

(*, Chi-squared test; †, Fisher's exact test)
